# Prevalence hearing loss of truck and bus drivers in a cross-sectional study of 65533 subjects

**DOI:** 10.1186/s12199-019-0831-7

**Published:** 2019-12-20

**Authors:** Siamak Pourabdian, Saeid Yazdanirad, Saeid Lotfi, Parastoo Golshiri, Behzad Mahaki

**Affiliations:** 1 Department of Occupational Health Engineering, School of Health, Isfahan University of Medical Sciences, Isfahan, Iran; 2 School of Health, Shahrekord University of Medical Sciences, Shahrekord, Iran; 3 Department of Occupational Health Engineering, School of Health, Tehran University of Medical Sciences, Tehran, Iran; 4 Department of Community Medicine and Family Physician, School of Medicine, Isfahan University of Medical Sciences, Isfahan, Iran; 5 Department of Biostatistics, School of Health, Kermanshah University of Medical Sciences, Kermanshah, Iran

**Keywords:** Hearing loss, Truck drivers, Bus drivers, Prevalence

## Abstract

**Background:**

Noise pollution is one of the most important occupational pollutants in heavy-vehicle drivers. Therefore, this epidemiological research was conducted with the aim of determining the prevalence of hearing loss in heavy-vehicle drivers in Iran.

**Methods:**

This cross-sectional research was conducted on 65,533 heavy-vehicle drivers including truck and intercity bus drivers from February 2006 to March 2016. The air and bone threshold of pure tone was measured for each ear at 0.5, 1, 2, 3, 4, 6, and 8 kHz by a skillful radiology expert. The obtained data from this research was analyzed in SPSS software using statistical tests such as descriptive analysis and paired *t* test.

**Results:**

Mean (standard deviation) of hearing loss in left and right ears of all people was 23.02 (8.25) and 22.48 (7.86), respectively. Paired *t* test showed that hearing loss difference in left and right ears was significant (*P* < 0.001). Mean and standard deviation of paired *t* test showed that hearing loss difference in left and right ears was significant in all frequencies except 1000 Hz (*P* < 0.001).

**Conclusion:**

The findings of this research generally showed that 26.8% of the studied drivers have hearing loss. Hearing loss in the left ear was more than right ear.

## Background

Noise pollution is one of the most important jobs and environmental pollutants [1], World Health Organization (WHO) has estimated the imposed damages by noise pollution to be about four million dollars [2]. The most important source of noise people hear on a daily basis is the one caused by vehicles. Committee of Europe (CE) stated that the noise pollution caused by cars is about 70–80 dB and busses about 80–95 dB [3]. Some studies have identified the sources of noise pollution. The most important factor of noise pollution in low speed vehicles is the power transfer system of the vehicle which includes air valves, engine noise, exhaust system, fan, air blower, filter, and eventually move up to the top axis [4]. Another factor in noise making includes vehicle tires. Tires are the major source of noise at speeds higher than 30–50 km/h [5]. Heavy vehicles produce more noise than light ones due to their higher weights based on the number of wheels [5]. Aerodynamics is the third source of noise production in vehicles. Airflow contacts with various parts of the body such as mirrors and columns cause noise emission. Aerodynamic factor becomes significantly important in speeds higher than 80 km/h [6].

The noise of the vehicle can influence various groups including dwellers around heavy traffic roads and vehicle drivers. Many studies have been conducted on the environmental effects of noise pollution. The national research in the US shows that 18% of people suffer from road traffic noise in their lives [7]. However, noise pollution and the resulting effects on the driver’s vehicle particularly intercity heavy-vehicle drivers have not really been mentioned. Experiencing noise at high levels can have various effects on human. Noise as an undesirable sound can have various effects such as hearing loss, sleep disorders, hypertension, coronary artery disease, and gastrointestinal ulcers [8, 9]. Meanwhile, hearing loss is one of the most important effects of noise. Hearing loss is caused by a sensory-neural damage develop during years of being faced with noise and it is preventive, but irreversible [10]. Hearing loss by noise occurs as a result of death of hair cells in ears and cochlear damage caused by metabolic changes in the body [11]. Hearing loss besides the direct effects can also cause disability in daily activities and lives of people [12]. It can even lead to job loss and mental effects [13].

According to the importance of preventing hearing loss in developing countries, bus and truck drivers are examined annually in Iran for hearing loss, but there is no comprehensive epidemiological study to analyze these findings. Therefore, this epidemiological research was conducted with the aim of determining the prevalence of hearing loss in heavy-vehicle drivers of Iran.

## Methods

This cross-sectional research was conducted on heavy-vehicle drivers including truck and intercity bus drivers from February 2006 to March 2016. This study is a part of a national survey program. Isfahan province, as one of the industrial cities with high traffic of heavy vehicles in center of Iran, was selected to perform the study. Therefore, all intercity drivers referred to occupational medical centers of Isfahan province to obtain a health card during these 10 years were included in the study. The date of the drivers extracted and the date of the subjects with the inclusion criteria entered to the study. Inclusion criteria include: older than 20 years, no other diseases except musculoskeletal disorders or back pain, no congenital hearing impairment and diagnosis of guided or combined hearing loss, no background of ear discharge, and no extra secretion or wax. For the collection of the driver’s data during the last 10 years, their demographic information including age, height, and weight was collected at first. Then, their general health was studied. Finally, they passed a pure turn audiometry. The data of 65533 drivers were collected in this period. All participants were male.

Pure tone air and bone conduction audiometry parameter was used to test the hearing condition of people. Hearing was tested using the Welton 1300 clinical audiometer equipped with AD-19 supra-aural in a noise-insulated room based on the American Speech-Language-Hearing Association [14, 15]. The air and bone threshold of pure tone was measured for each ear at 0.5, 1, 2, 3, 4, 6, and 8 kHz by a skillful radiology expert. The environmental noise level was so low in the test room that it did not allow the hearing loss test even to 0 dB connecting air phone to the tester. Audiometer and all the relevant equipment were calibrated before starting the research and every 3 months.

Hearing loss was calculated as mean threshold pure tune at 0.5, 1, 2, 3, 4, 6, and 8 kHz in each ear. Classification of people was based on hearing loss as people with healthy ears (HL less than 25), people with weak hearing loss (higher HL than 25 and similar/lower than 40), people with medium hearing loss (HL higher than 40 and similar/lower than 60), and people with high hearing loss (higher than 60) [15]. Moreover, people with higher than 25 dB hearing loss were studied just in one of these frequencies and were considered as those with hearing loss. Ethical approval was obtained from the local research ethics committee to conduct this research.

The obtained data from this research was analyzed in SPSS software using statistical tests such as descriptive analysis and paired *t* test. Paired *t* test was used to evaluate the difference of hearing loss between the right and left ears. *P* value below 0.05 was considered as statistically significant.

## Results

Mean (standard deviation), age, height, weight, and body mass index of the participants were 38.2 (12.2), 1.73 (0.06), 76.69 (12.95), and 25.58 (9.64). All participants were male. The results of statistical analysis showed that 47,998 out of 65,533 (73.2%) were without significant hearing loss, 2891 (4.4%) with hearing loss just in right ear, 5081 people (7.8%) with hearing loss just in the left ear, and 9563 (14.6%) had hearing loss in both ears. Frequency and frequency percentage of people with various hearing loss degrees in the left and right ears are presented in Table 1. Mean (standard deviation) of hearing loss in left and right ears of all people was 23.02 (8.25) and 22.48 (7.86), respectively. In addition, mean (standard deviation) of hearing loss in left and right ears of those affected with hearing loss was 36.84 (10.32) and 35.78 (10.19), respectively. Paired *t* test showed that hearing loss difference in the left and right ears was significant (*P* < 0.001). As well, Table 2 reports mean and standard deviation of the hearing loss in the left and right ears at the different frequencies. In addition, the results of the hearing loss difference in the left and right ears evaluated by paired *t* test in all frequencies have been presented in Table 2. Based on the results, most values of hearing loss in the left ear equal to 26.18, 25.96, and 25.60 dB were related to the frequencies of 8000, 6000, and 4000 Hz, respectively. As well, the frequencies of 8000, 6000, and 4000 Hz had most values of hearing loss in the right ear equal to 25.26, 25.06, and 24.75, respectively.

**Table 1:** Frequency and percent of subjects with different degrees of hearing loss in the right and left ears

		Different degrees of hearing loss in the right ear				
		Without hearing loss	Mild	Moderate	Severe	Total
Different degrees of hearing loss in the left ear	Without hearing loss	47998 (73.7 %)	2395 (3.7 %)	286 (0.4 %)	71 (0.1 %)	50750 (77.9 %)
	Mild	4507 (6.9 %)	6052 (9.3 %)	664 (1.0 %)	78 (0.1 %)	11301 (17.4 %)
	Moderate	386 (0.6 %)	1027 (1.6 %)	1133 (1.7 %)	107 (0.2 %)	2653 (4.1 %)
	Severe	76 (0.1 %)	113 (0.2 %)	106 (0.2 %)	123 (0.2 %)	418 (0.6 %)
	Total	52967 (81.3 %)	9587 (14.7)	2189 (3.4 %)	379 (0.6 %)	65122 (100 %)

**Table 2:** Mean and standard deviation of the hearing loss in the left and right ears at the different frequencies

Frequency (Hz)	Hearing loss in the left ear		Hearing loss in the right ear		*P* value^*^
	Mean	Standard deviation	Mean	Standard deviation	
500	20.08	5.28	19.93	5.26	*P* < 0.001
1000	19.39	5.47	19.36	5.63	0.106
2000	20.75	7.07	20.40	6.56	*P* < 0.001
3000	22.91	10.29	22.30	9.63	*P* < 0.001
4000	25.60	13.07	24.75	12.53	*P* < 0.001
6000	25.96	13.77	25.06	12.96	*P* < 0.001
8000	26.18	14.67	25.26	13.82	*P* < 0.001

^*^P values were calculated by paired *t* test

**Fig. 1:**
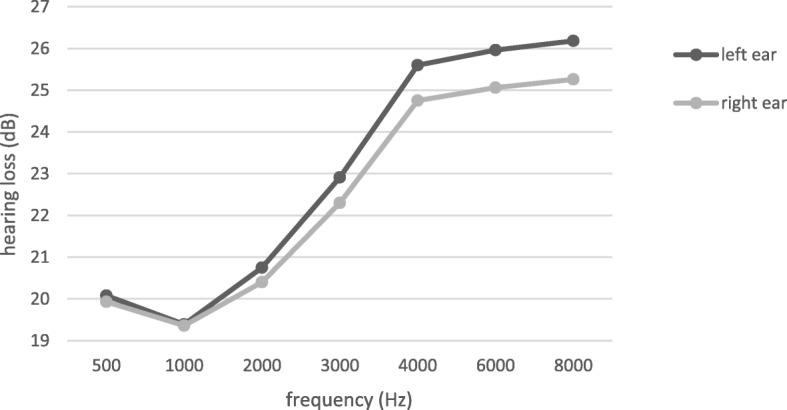
Hearing loss in the left and right ears at the different frequencies

The results of paired *t* test also indicated that difference of mean hearing loss between left and right ears was significant in all frequencies except 1000 Hz (*P* < 0.001). Figure 1 shows the hearing loss in both left and right ears in various frequencies. The results of Table 2 and Fig. 1 revealed that differences of hearing loss value between the right and left ears in high frequencies (4000, 6000, and 8000 Hz) were more than that in lower frequencies.

## Discussion

Results of this research generally showed that 26.8% of drivers had hearing loss. Meanwhile, the maximum percentage (14.6%) is related to people with hearing loss in both ears. Among those who had hearing loss just in one ear, hearing loss in the left ear was 7.8% more than the ones with hearing loss in the right ear with 4.4%. Janghorbani et al. in a research with the aim of estimating the prevalence of hearing loss in truck drivers concluded that the prevalence of hearing loss in both ears, in the left ear, and in the right ear among drivers was 18.1, 6.5, and 3%, respectively [16]. Results of this research are relatively in line with the results of Janghorbani research results. Nevertheless, some differences in findings is because Janghorbani’s research was conducted for 4300 persons, while his research was conducted on 65533 persons. According to the results of Nelson et al., 16% of disabling hearing loss in adults is attributed to noise in the place of work which is in the range of 7–21% in various groups [17]. The prevalence of hearing loss due to a noisy job in this research about truck drivers can be attributed to the different definitions of hearing loss, used methodology, and the difference in access to medical cares.

Moreover, the high volume of the decrepit trucks and busses with high noise in Iranian roads can also be a reason for this finding. Head of Iran's Road and Rail Transport Organization in 2017 announced that there are 252000 trucks younger than 15 years, 96000 trucks of 15–35 years old, and 75000 trucks older than 35 years working in the state [18]. Another reason for drivers hearing loss can also be due to high consumption of cigarette and drugs in this regard. Therefore, some studies show that cigarette abuse has an important role in causing a double hearing loss and can result from noise [19, 20]. Anyway, it seems that the prevalence of hearing loss among drivers is similar to other countries all over the world while facing high noise levels [17].

Moreover, results of this research showed that the mean hearing loss in Iranian drivers in left and right ears is 23.02 and 22.48. Results of Krishman Kumar and Jain research in India show that noise pressure level of trucks and busses is 83-90 and 77-92 dB, respectively [21]. Results of Soltanzadeh et al. review on studies in Iran in 1997-2012 show that the mean level of noise pressure in Iran jobs is 90.29 dB and mean hearing loss is 26.44 dB [22]. This value of hearing loss is close to the values of hearing loss in this research. Furthermore, results of this research showed that hearing loss of drivers in left ear is more than right ear. In addition, the number of people affected with left ear hearing loss particularly with medium and high degree was more than those with right ear hearing loss. These findings are in line with the findings of other published studies [23, 24]. These results were obtained because drivers pull down their vehicles' windows to gain access to fresh air and cool environment instead of using air conditioner. The number of decrepit heavy vehicles is high in Iran and some of them don’t even have an air conditioner. The main noise resources in automobile included engine, road/tire, aerodynamic air flow, and exhaust that are outside of the truck chamber [25]. In addition, wind flow because of open windows also is one of important noise resources [25]. Therefore, when the driver's window placed on the left side is open or partially open, the noise due to these sources enters the chamber and affects the hearing loss, particularly in left ear that is more near to the window. In addition, results of this research showed that the difference in hearing loss in left and right ears was significant in all frequencies except 1000 Hz, and this difference increased as frequency increases. Hearing loss in the left and right ears was relatively similar in 4000, 6000, and 8000 frequencies. The result of Nandi et al.’s research with the aim of reviewing the resulting hearing loss studies in India shows that the resulting hearing loss is usually mutual and usually influence the higher frequencies (3, 4, and 6 kHz); then, it develops to the lower frequencies (0.5, 1, and 2 kHz) [26].

According to the high volume of samples in this research, the limitations of this research can be the non-consideration of people’s working background, the exact type of vehicle model, and the condition of its air conditioning system. Moreover, the studied blood parameters are fewer than the other research and more blood parameters can be evaluated in future research.

## Conclusion

The findings of this research generally showed that 26.8% of the studied drivers have hearing loss. Hearing loss in the left ear was more than that in the right ear. In addition, people with hearing loss just in left ear were more than those just in the right ear. According to the hearing importance of drivers’ safety and life quality, it is suggested to reduce people facing this noise by innovation of the decrepit trucks, installation of suitable ventilation systems on old trucks to avoid opening the windows, isolation of the holes and vents of the truck chamber, periodic maintenance of trucks, use of personal protective equipment, decrease of driving time duration, and periodic health check for early detection of individuals with hearing loss and work restrictions for them to prevent further progression.

## Data Availability

All data generated and analyzed during this study are included in this published article.

## Abbreviation

WHO: World Health Organization
CE: Committee of Europe
Hz: Hertz
